# Investigation of a new acetogen isolated from an enrichment of the tammar wallaby forestomach

**DOI:** 10.1186/s12866-014-0314-3

**Published:** 2014-12-11

**Authors:** Emma J Gagen, Jiakun Wang, Jagadish Padmanabha, Jing Liu, Isabela Pena Carvalho de Carvalho, Jianxin Liu, Richard I Webb, Rafat Al Jassim, Mark Morrison, Stuart E Denman, Christopher S McSweeney

**Affiliations:** CSIRO Agriculture, St Lucia, Australia; School of Agriculture and Food Sciences, The University of Queensland, Gatton, Australia; Key Laboratory of Molecular Animal Nutrition, Ministry of Education, Zhejiang University, Hangzhou, China; Faculty of Agricultural & Veterinary Sciences, Universidade Estadual Paulista, Campus de Jaboticabal, Brazil; Centre for Microscopy and Microanalysis, The University of Queensland, St Lucia, Australia

**Keywords:** Acetogen, Acetogenesis, Tammar wallaby, Rumen, Methanogenesis

## Abstract

**Background:**

Forestomach fermentation in Australian marsupials such as wallabies and kangaroos, though analogous to rumen fermentation, results in lower methane emissions. Insights into hydrogenotrophy in these systems could help in devising strategies to reduce ruminal methanogenesis. Reductive acetogenesis may be a significant hydrogen sink in these systems and previous molecular analyses have revealed a novel diversity of putative acetogens in the tammar wallaby forestomach.

**Results:**

Methanogen-inhibited enrichment cultures prepared from tammar wallaby forestomach contents consumed hydrogen and produced primarily acetate. Functional gene (formyltetrahydrofolate synthetase and acetyl-CoA synthase) analyses revealed a restricted diversity of *Clostridiales* species as the putative acetogens in the cultures. A new acetogen (growth on H_2_/CO_2_ with acetate as primary end product) designated isolate TWA4, was obtained from the cultures. Isolate TWA4 classified within the *Lachnospiraceae* and demonstrated >97% *rrs* identity to previously isolated kangaroo acetogens. Isolate TWA4 was a potent hydrogenotroph and demonstrated excellent mixotrophic growth (concomitant consumption of hydrogen during heterotrophic growth) with glycerol. Mixotrophic growth of isolate TWA4 on glycerol resulted in increased cell densities and acetate production compared to autotrophic growth. Co-cultures with an autotrophic methanogen *Methanobrevibacter smithii* revealed that isolate TWA4 performed reductive acetogenesis under high hydrogen concentration (>5 mM), but not at low concentrations. Under heterotrophic growth conditions, isolate TWA4 did not significantly stimulate methanogenesis in a co-culture with *M. smithii* contrary to the expectation for organisms growing fermentatively.

**Conclusions:**

The unique properties of tammar wallaby acetogens might be contributing factors to reduced methanogen numbers and methane emissions from tammar wallaby forestomach fermentation, compared to ruminal fermentation. The macropod forestomach may be a useful source of acetogens for future strategies to reduce methane emissions from ruminants, particularly if these strategies also include some level of methane suppression and/or acetogen stimulation, for example by harnessing mixotrophic growth capabilities

**Electronic supplementary material:**

The online version of this article (doi:10.1186/s12866-014-0314-3) contains supplementary material, which is available to authorized users.

## Background

Methane is a potent greenhouse gas that has been implicated in global warming [1]. Enteric fermentation of ruminant livestock is the largest source of anthropogenic methane, contributing between 20 and 25% of global methane emissions [2]. During ruminal fermentation, methanogenic archaea use hydrogen to reduce carbon dioxide or methylated compounds to methane, contributing to global methane emissions and representing a loss of between 2 and 12% ingested feed energy for the ruminant [3]. Australian macropod marsupials such as kangaroos and wallabies exhibit foregut fermentation analogous to ruminants but resulting in lower methane emissions [4-6], which suggests either lower hydrogen production from foregut fermentation or the functioning of alternative hydrogen disposal mechanisms to methanogenesis in these animals. If the latter, understanding hydrogenotrophy in these foregut systems may provide insight into mechanisms for redirecting hydrogen away from methanogenesis in ruminants. Acetogenesis (4H_2_ + 2CO_2_ → CH_3_COOH +2H_2_O) may represent a significant hydrogen sink in the foregut of native Australian marsupials and these animals may be a source of novel acetogens [7-10]. Recent molecular analyses revealed a diverse and novel population of putative acetogens in the forestomach of the tammar wallaby that was significantly different to the population present in ruminants [8]. Potentially, acetogen population differences between native Australian marsupials and ruminants is causal or results from reduced methane emissions from foregut fermentation in these animals. The aims of the present study were to investigate the novel acetogen diversity from the tammar wallaby forestomach using enrichment cultures and molecular analysis of enrichment cultures, as well as to isolate acetogens from the tammar wallaby forestomach and investigate factors that may stimulate their hydrogenotrophic capacities. We report here the isolation of a novel acetogen, isolate TWA4 from the tammar wallaby forestomach and its hydrogenotrophic capacity, mixotrophic growth capabilities and interactions with a methanogen in co-culture under autotrophic and heterotrophic growth conditions. We also report on partial genomic information from isolate TWA4 relating to the Wood-Ljungdahl pathway genes and to genes for glycerol metabolism.

## Results

### Characterisation of tammar wallaby forestomach acetogen enrichment cultures *Hydrogen consumption and acetate production*

Acetogen enrichment cultures from tammar wallaby forestomach contents consumed net hydrogen and carbon dioxide. Acetic acid was the major end product detected, a small amount of sulphide and minor amounts of other acids were also produced and no methane was detected (Additional file 1). Acetate production accounted for 61 ± 2.3% of hydrogen consumed based on stoichiometry of the acetyl-CoA pathway (H_2_ consumption:acetate production ratio for enrichment cultures was 6.58 ± 0.256).

### Molecular analysis of tammar wallaby forestomach acetogen enrichment cultures

The 16S rRNA gene (*rrs*) library (n = 96) from tammar wallaby forestomach acetogen enrichment cultures revealed a restricted bacterial diversity (9 OTUs) and rarefaction analysis indicated that an acceptable level of sequencing coverage had been achieved (Additional file 2). One OTU from the cultures showed > 97% *rrs* identity to *Enterococcus faecium*, another contained sequences that demonstrated > 97% *rrs* identity to recently isolated kangaroo acetogens YE273, YE266 and YE257 that are yet to be formally classified [10] and the remaining OTUs were classified as *Clostridiales* or *Tenericutes* sequences and did not demonstrate > 97% sequence identity to any named species. The *rrs* from an isolate later obtained from the cultures (isolate TWA4) clustered within the OTU demonstrating > 97% identity to kangaroo acetogens (Additional file 3).

FTHFS sequences (n = 64) from tammar wallaby forestomach enrichments clustered into four OTUs that affiliated with the *Clostridiaceae* and demonstrated homoacetogen similarity (HS) scores > 90% (Additional file 4a). Rarefaction analysis indicated that an acceptable level of coverage had been achieved for FTHFS sequences (Additional file 4b). Sequences in one FTHFS OTU (OTU 2, Additional file 4a) were the same as a sequence detected in the tammar wallaby previously [8]. The two largest FTHFS OTUs from the enrichments grouped with two copies of FTHFS recovered from isolate TWA4, which was later isolated from the cultures (Additional file 4a). ACS sequences (n = 41) from the enrichment cultures also clustered into four OTUs, one identical to an ACS from *Blautia hydrogenotrophica* and detected in the tammar wallaby previously [8], the others affiliating with the *Lachnospiraceae* or forming a sister group to the *Clostridiaceae*. Sequences in the two largest ACS OTUs from the enrichments were identical to two ACS sequences recovered from isolate TWA4, later isolated from these enrichments (Additional file 5a); one of these sequences had been reported in the tammar wallaby previously [8]. Rarefaction analysis indicated that an acceptable level of coverage had been achieved for ACS sequences (Additional file 5b).

### Isolation and preliminary characterisation of acetogens from the tammar wallaby forestomach

Three isolates in pure culture that were obtained from tammar wallaby forestomach enrichment cultures were considered potential acetogens after demonstrating the presence of *fhs* and showing net H_2_ and CO_2_ consumption and producing acetate as the major end product when grown on modified AC11.1 medium with H_2_:CO_2_ provided. Detailed analyses were only performed on one isolate, hereafter referred to as isolate TWA4. Isolate TWA4 was a strict anaerobe. Phylogenetically, isolate TWA4 classified within Cluster XIV of the *Clostridium* sub-phylum, within the family *Lachnospiraceae* and demonstrated > 97% *rrs* identity to three recently isolated kangaroo acetogens: strains YE273, YE266 and YE257 (Additional file 3). The nearest named isolate to these species was *Ruminococcus gnavus*, which demonstrated only 92.35% *rrs* identity to isolate TWA4. The nearest named rumen acetogen was *Acetitomaculum ruminis* which demonstrated 88.52% identity to isolate TWA4 (Additional file 3). Two copies of each of the key reductive acetogenesis genes *acsB* and *fhs* were recovered from isolate TWA4. The FTHFS sequences showed ~93.9% amino acid similarity to each other and both affiliated with the *Clostridiaceae* (sequences TWA4_1 and TWA4_2, Additional file 4a). The ACS sequences demonstrated only 63.38% amino acid identity to each other and one copy affiliated with the *Clostridiaceae* while the second copy of *acsB* affiliated distantly with the *Lachnospiraceae* (sequences TWA4_1 and TWA4_2, Additional file 5a).

### Morphology and cell structure of tammar wallaby isolate TWA4

**Figure 1:**
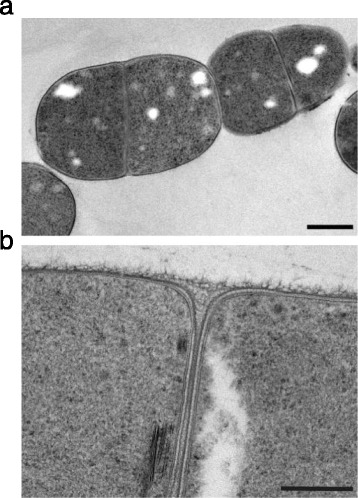
**Transmission electron micrographs of isolate TWA4.** Isolate TWA4 grown on **(a)** modified AC11.1 medium with H_2_:CO_2_ and 10 mM glycerol and **(b)** modified AC11.1 medium with H_2_:CO_2_ only. Scale bar represents **(a)** 500 nm and **(b)** 200 nm.

Isolate TWA4 grew as straight rods, varying in length from 1.0 to 2.8 μm and diameter from 0.7 to 2.0 μm (Figure 1a). Cells mainly occurred in pairs or sometimes in clusters or short chains of three to five cells. Electron microscopy revealed a typical Gram positive cell wall structure for TWA4 (Figure 1b), while staining properties and cellular morphology varied with culture conditions. For example, when glycerol was included in the culture medium, TWA4 cells displayed white, electron-luscent dense areas (Figure 1a), that were not present in cells grown on medium without glycerol (Figure 1b). Also, compared to TWA4 cells grown without glycerol, cells grown with glycerol displayed a smaller amount of capsular material and were more prone to forming short chains. Short stacks of membranes were frequently seen in cells grown without glycerol (Figure 1b) but never when glycerol was present in the medium.

### Metabolic and mixotrophic capabilities of tammar wallaby isolate TWA4

**Figure 2:**
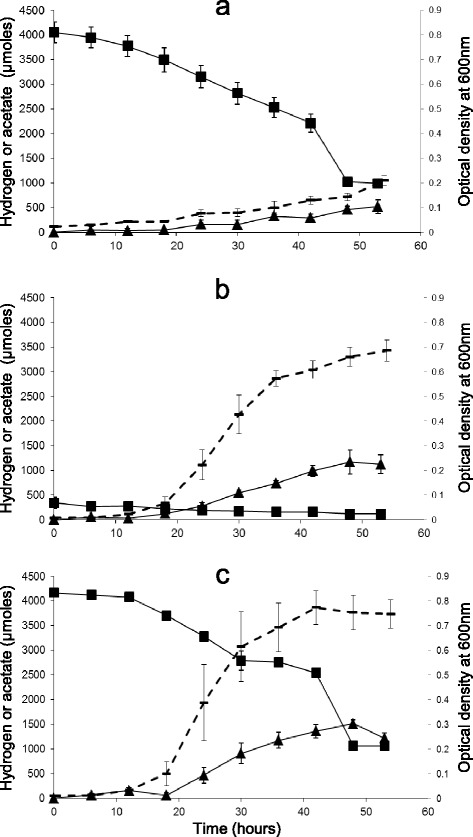
**Hydrogen consumption, acetate production and growth of isolate TWA4 under autotrophic, heterotrophic and mixotrophic conditions.** Isolate TWA4 grown on modified AC11.1 medium with **(a)** H_2_:CO_2_ only **(b)** 10 mM glycerol and no overpressure of H_2_:CO_2_ and **(c)** 10 mM glycerol and H_2_:CO_2_ provided. Hydrogen is represented by squares, acetate by triangles and optical density by a dashed line. Error bars represent standard error of mean (n = 3).

When grown on modified AC11.1 medium, isolate TWA4 consumed net hydrogen (3206 ± 156 μmoles) and carbon dioxide (1745 ± 58 μmoles) with acetate (523 ± 141 μmoles) as the major end product (Figure 2a). The maximal cell density under these conditions as measured by OD_600_ was ~0.2 (Figure 2a). There was large variability in the hydrogen consumption:acetate production ratios of individual cultures in this experiment, possibly as a result of subsampling gases and short chain fatty acids (SCFAs) every 6 hours throughout the culture progression. When cultures of isolate TWA4 were not disturbed during incubation and gases and SCFAs were measured only at end point, hydrogen consumption:acetate production ratios of 5.56 ± 0.263 were observed.

**Figure 3:**
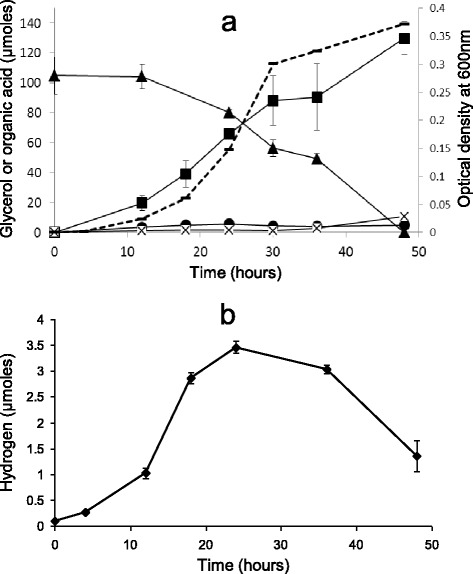
**Substrate consumption and end product formation by isolate TWA4 under heterotrophic conditions. (a)** Glycerol (triangles) consumption, acetate (squares), formic acid (circles) and citrate (crosses) production and culture optical density (dashed line) and **(b)** hydrogen profile of isolate TWA4 when grown on modified AC11.1 medium with 10 mM glycerol as the substrate and no exogenous hydrogen provided. Error bars represent standard error of mean (n = 3).

Glycerol was found to be stimulatory to the growth and acetate production of isolate TWA4 with hydrogen consumed concomitantly, indicating mixotrophy. Over a 48 hour period, isolate TWA4 produced significantly more acetate and higher cell densities when grown on medium with glycerol alone (p < 0.05) or with glycerol plus H_2_:CO_2_ (p < 0.001), than when grown on medium with only H_2_:CO_2_ available as substrate (Figure 2). Hydrogen was consumed by isolate TWA4 during growth under all three conditions and hydrogen consumption was not significantly different between medium with only H_2_:CO_2_ available as substrates and medium with both glycerol and H_2_:CO_2_ provided (p > 0.05, see Figure 2a and c). Hydrogen did not accumulate in significant amounts during growth of TWA4 on glycerol alone and the residual hydrogen present in the headspace of culture from medium preparation in an anaerobic chamber, was consumed (Figure 2b). During growth on glycerol alone, glycerol was consumed within 48 hours, acetate was the major end product (glycerol consumption to acetate production ratios were ~ 0.81), minor amounts of formate and citrate were also produced (Figure 3a) and 1,3-propanediol and other alcohols were not detected. During growth on glycerol alone, hydrogen accumulated to a maximum of 3.46 ± 0.11 μmoles (in a 17 ml headspace, ~4620 ppm hydrogen) within 24 hours and thereafter declined to 1.35 ± 0.29 μmoles over the next 24 hours (Figure 3b).

### Co-culture of isolate TWA4 with a methanogen

**Figure 4:**
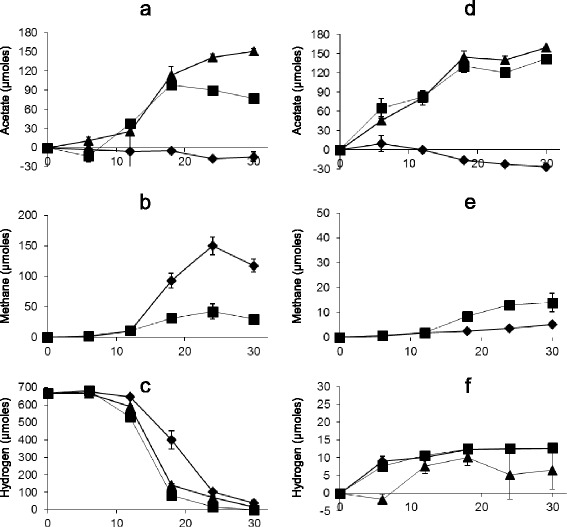
**Acetate, methane and hydrogen profiles of isolate TWA4 and a methanogen autotrophic and heterotrophic conditions.** Acetate, methane and hydrogen profiles of isolate TWA4 (triangles), *M. smithii* (diamonds) or co-cultures of TWA4 and *M. smithii* (squares) during growth on modified AC11.1Y medium with only H_2_:CO_2_ provided as substrates (panels **a, b** and **c**) or on modified AC11.1Y medium with 10 mM glycerol and without exogenous hydrogen provided (panels **d, e** and **f**). Error bars represent standard error of mean (n = 3).

Cultures of *M. smithii* and isolate TWA4 when grown together under H_2_:CO_2_ consumed net hydrogen to produce both acetate and methane as end products, indicating successful growth of both strains. During the first 18 hours of growth, the pattern of hydrogen consumption and acetate production in co-cultures followed that observed for isolate TWA4 alone (Figure 4a, c), while methane production was significantly (p <0.01) reduced across this period for co-cultures compared to cultures of *M. smithii* alone (Figure 4b). However, by 18 hours the hydrogen concentration in the headspace of culture tubes declined to approximately 82 μmoles (in a 17 ml headspace, ~4.8 mM), no further acetate production was observed and the remaining hydrogen was consumed and methanogenesis started to dominate acetogenesis (Figure 4a-c).

In co-cultures of isolate TWA4 and *M. smithii* without H_2_:CO_2_ provided but with glycerol as a substrate (Figure 4d-f), acetate production was not significantly different to that observed by isolate TWA4 alone (p > 0.05) and the minimal methane that was produced was not significantly different (p = 0.08) to that observed in pure cultures of *M. smithii* without an overpressure of H_2_:CO_2_ in the headspace (i.e. with only the residual hydrogen from the anaerobic chamber atmosphere in culture headspaces).

### Genomic analysis of isolate TWA4 with respect to the acetyl-CoA pathway and potential glycerol metabolism pathways

Sequence reads assembled using Newbler (v 2.6) generated eight scaffolds with an estimated genome size of 3.4 Mb at 12 times coverage. 98.69% of the reads and 85.36% of the contigs were assembled into the largest scaffold of just over 3.1 Mb. The average gap in the assembled data was calculated as 870 bp. Orphan contigs greater than 500 bp and scaffolds produced 3.2 Mb of data that was comprised of 3038 open reading frames of which 34% were of unknown function.

**Figure 5:**
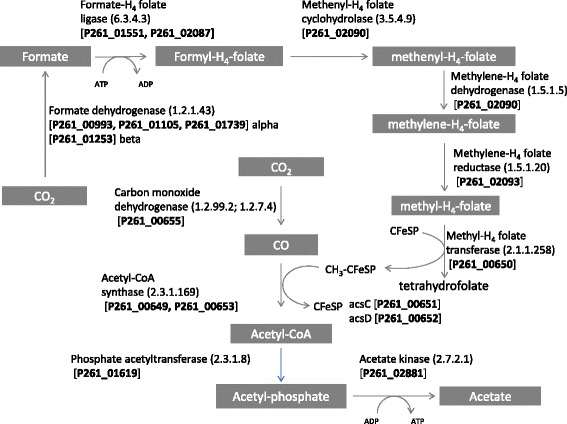
**The Wood-Ljungdahl pathway of reductive acetogenesis for isolate TWA4.** Predicted genes encoding steps of the pathway are shown with their respective locus tag.

**Figure 6:**
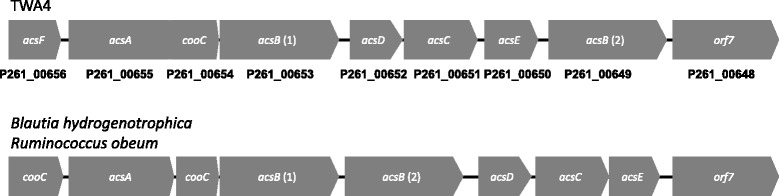
**Arrangement of the Wood-Ljungdahl core gene cluster for isolate TWA4,** ***Blautia hydrogenotrophica*** **and** ***Ruminococcus obeum.*** *cooC*: CODH chaperone; *acsA*: CODH; *acsB*: ACS; *acsD*: CFeSP alpha subunit; *acsC*: CFeSP beta subunit, *acsE*: methyltransferase; *orf7*: ferrodoxin.

**Figure 7:**
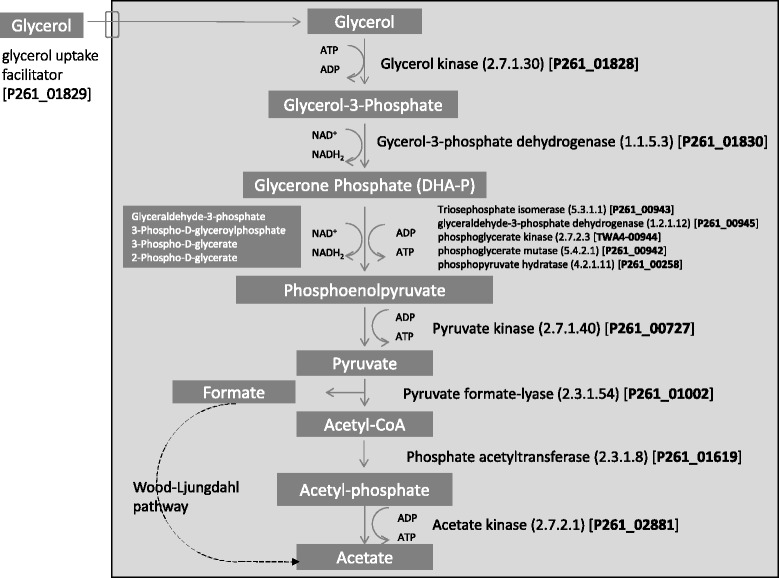
**The metabolic pathway for the fermentation of glycerol for isolate TWA4.** Predicted genes encoding steps of the pathway are shown with their respective locus tag.

Genes encoding the enzymes of the complete acetyl-CoA pathway were present in the genome of isolate TWA4 (Figure 5) and key genes of this pathway clustered together (Figure 6) with a gene order that did not resemble any of the five groups of Wood-Ljungdahl pathway gene clusters reported to date [11]. Genes encoding enzymes for the degradation of glycerol to acetate via glycerol-3-phosphate were found in the genome of isolate TWA4 (see Figure 7) and genes encoding the enzymes glycerol dehydrogenase (EC 1.1.1.6) and dihydroxyacetone kinase (EC 2.7.1.29) which are responsible for conversion of glycerol to dihydroxyacetone-phosphate via dihydroxyacetone, were not evident in the genome of isolate TWA4 [e.g. for an overview of glycerol degradation pathways see [12]].

## Discussion

In order to fully understand complex microbial ecosystems and/or harness microbial activities for any useful purpose, it is essential that researchers combine the strengths of both cultivation and molecular-based techniques. In the present study we employed a combination of cultivation and molecular approaches to successfully enrich for acetogens from tammar wallaby forestomach contents, which had previously only been investigated by molecular methods [8]. Molecular analysis of enrichment cultures in the present study revealed novel putative acetogens with functional genes affiliating between the *Lachnospiraceae* and *Clostridiaceae* and not close to sequences from any previously known acetogen (Additional files 4a and 5a). We successfully isolated one of these novel acetogens (isolate TWA4) which classified within the *Lachnospiraceae* and may belong to the same species as recently isolated acetogens from the forestomach of eastern grey (*Macropus giganteus*) and red kangaroos (*Macropus rufus*) [10].

Isolate TWA4 was a potent hydrogenotroph rapidly consuming exogenous hydrogen and carbon dioxide with acetate as the sole or primary end product. Hydrogen consumption:acetate ratios for isolate TWA4 were at times higher than expected based on stoichiometry of the acetyl-CoA pathway of reductive acetogenesis (4H_2_ + 2CO_2_ → CH_3_COOH +2H_2_O), most likely due to consumption of hydrogen and carbon dioxide for formation of cell carbon and capsular material for new biomass, as end products other than acetate were not detected. Genomic analysis confirmed the presence of all genes for the Wood-Ljungdahl acetyl-CoA pathway of reductive acetogenesis in isolate TWA4 (Figure 5). However, the order of key genes of this pathway in the isolate TWA4 genome was not consistent with any of the previously reported gene orders for other microorganisms possessing this pathway [11]. The order of key Wood-Ljungdahl pathway genes in TWA4 was remarkably similar to that of two other species whose genome has recently been completed, *Blautia hydrogenotrophica* and *Ruminococcus obeum* (Figure 6) and we propose that this ordering constitutes a new gene order group. Though this gene ordering is most similar to the gene order of what is currently known as group C [11], isolate TWA4, *B. hydrogenotrophica* and *R. obeum* all show replacement of the formyl tetrahydrofolate synthase, the bifunctional formyltetrahydrofolate cyclohydrolase/dehydrogenase and methylene tetrahydrofolate reductase cluster of group C with a second *acsB* copy (Figure 6). Multiple copies of *fhs* and *acsB* have been detected in other pure culture acetogens using PCR-based methods previously [8]. The presence of two *acsB* and *fhs* in isolate TWA4, which match to the majority of *acsB* and *fhs* sequences recovered from tammar wallaby acetogen enrichment cultures (Additional files 4a and 5a), indicate that isolate TWA4 was the dominant acetogen enriched for in the culture approach used in this study. Functional genes from strain TWA4 have been recovered from the tammar wallaby forestomach previously [8] indicating that this species forms a substantial proportion of the acetogen population in the tammar wallaby forestomach naturally and is detectable without any selection or cultivation bias. However, these functional genes were not dominant within FTHFS and ACS libraries from the tammar wallaby forestomach naturally [8] suggesting that other, potentially major, acetogens are present in vivo and were not selected for with the cultivation conditions used in the present study.

In addition to autotrophic growth, isolate TWA4 demonstrated excellent mixotrophic capabilities, consuming hydrogen just as rapidly during growth on glycerol and H_2_:CO_2_ as on H_2_:CO_2_ alone and producing significantly more acetate and more than twice the cell mass as a result of mixotrophy. Mixotrophy influences the competitiveness of acetogens over methanogens in some ecosystems [13] and has been suggested as an important trait required for any acetogen that will be used in strategies for reducing ruminal methanogenesis [14,15]. While many acetogens are capable of mixotrophic growth, to date only one rumen acetogen with this ability has been reported [14]. It is generally thought that most rumen acetogens growing in the presence of carbohydrates will ferment the available substrate rather than grow autotrophically or mixotrophically (e.g. as for strain ser8 in [14]) and in a mixed community they would therefore make hydrogen available for interspecies transfer [16] to methanogens. Our investigations of isolate TWA4 in pure culture and in co-culture with a methanogen do not support this idea. In contrast to typical fermentative growth on glycerol (e.g. for *Thermotoga* spp.) which results in accumulation of acetate, CO_2_ and H_2_ [17] isolate TWA4 did not accumulate hydrogen as an end product. Rather, hydrogen generated during fermentation was consumed (Figure 3b), suggesting that isolate TWA4 was able to internally shuttle or utilise most of the reducing equivalents generated during fermentation. Furthermore, when isolate TWA4 was grown heterotrophically with glycerol as a substrate, in co-culture with a methanogen, methanogenesis was not significantly stimulated (p =0.08). Further experiments are necessary to confirm this finding with other methanogens and over longer growth periods, however it seems possible from these data that there may be potential to find appropriate acetogens to serve as alternative hydrogenotrophs in the rumen ecosystem in the future.

Although isolate TWA4 successfully performed reductive acetogenesis in the presence a hydrogenotrophic methanogen when hydrogen was available at high concentrations (Figure 4), isolate TWA4 was not able to compete with a methanogen for hydrogen when hydrogen concentrations in the headspace were low (in this case < 5 mM, Figure 4), as expected based on the energetics of reductive acetogenesis and methanogenesis [18,19]. These findings are in agreement with the available literature which indicates that autotrophic acetogenesis alone will not dominate over methanogens under natural rumen conditions (e.g. see [18]). However, potentially acetogen stimulation (e.g. by harnessing mixotrophic capabilities) along with methanogen suppression could be a useful strategy to establishing reductive acetogenesis as an alternative hydrogen sink in the rumen.

## Conclusions

Acetogens previously identified from the tammar wallaby forestomach by molecular methods were successfully enriched in culture. An acetogen obtained from these cultures, isolate TWA4, was a potent hydrogenotroph and capable of mixotrophic growth with glycerol. Under heterotrophic conditions with glycerol, isolate TWA4 did not significantly stimulate methane production by a co-cultured methanogen, suggesting that isolate TWA4 might be able to internally recycle some of the reducing equivalents it generates during fermentation. While more detailed experiments with other methanogens and growth conditions are necessary to support our findings, the unique properties of tammar wallaby acetogens that we have observed could potentially be contributing factors to reduced methanogenesis and methanogen numbers in the tammar wallaby forestomach compared to the rumen. The macropod forestomach may be a useful source of acetogens for future strategies to reduce methane emissions from ruminants if these strategies also include some level of methane suppression and/or acetogen stimulation for example by harnessing mixotrophic growth capabilities (e.g. [15,20-23]).

## Methods

### Sample collection

Forestomach contents were collected from three euthanized female tammar wallabies from a captive colony that had been grazing pasture and receiving a commercial grain pellet mix supplement, as reported previously [24,25]. The animals were sampled in November 2006 and at the time had free range access to pastures composed predominantly of Timothy Canary grass (*Phalaris angusta*) and were also provided with a commercial pellet mix containing wheat, bran, pollard, canola, soy, salt, sodium bicarbonate, bentonite, lime and a vitamin premix (Young Stockfeeds, NSW, Australia). A CSIRO Animal Ethics and Experimentation Committee approved euthanasia and tissue collection from the sample animals; animals were euthanized with an overdose of pentobarbitone sodium (CSIRO Sustainable Ecosystems Animal Ethics Approval Number 06–20). Prior to shipping to the laboratory on dry ice and subsequent storage at −80°C, forestomach contents were transferred to sterile containers containing a glycerol based cryoprotectant as outlined by McSweeney et al. [26].

### Enrichment cultures for potential acetogens

Enrichment culture medium was modified from the AC11.1 medium of Greening and Leedle [27] and contained per litre, 38 ml of Mineral solution 2, 38 ml of Mineral solution 3, 200 ml of clarified rumen fluid, 1 ml of Pfennigs trace element solution, 1 ml of haemin solution and 1 ml of resazurin indicator as outlined by McSweeney et al. [26] as well as 1.7 ml acetic acid, 0.6 ml propionic acid, 0.4 ml n-butyric acid, 0.1 ml of each of n-valeric, *iso*valeric, *iso*butyric and 2-methylbutyric acid, 2 g of Na_2_HPO_4_.2H_2_O, 6 g of NaHCO_3_, 0.5 g of yeast extract and 0.25 g of cysteine-HCl. The final pH was adjusted to between 6.4 and 6.8. All media were prepared using standard anaerobic techniques [28], were dispensed in an anaerobic chamber with an atmosphere of 95% CO_2_ and 5% H_2_ (COY Laboratory Products Inc., Ann Arbor, MI) and were sterilised by autoclaving at 121°C, 100 kPa for 20 minutes. Modified AC11.1 medium was dispensed either as 9.9 ml aliquots in 27 ml Balch tubes (Bellco, Vineland, NJ) or as 100 ml aliquots in 125 ml serum bottles, both sealed with butyl rubber stoppers (Bellco). Immediately before sample inoculation for enrichment cultures, bromoethane sulfonate (BES; Sigma, Australia) to final concentration 2 mM was added to modified AC11.1 medium to inhibit growth of methanogens.

Glycerol stocks of samples were thawed and approximately 0.1 ml of stored forestomach contents from individual animals were inoculated separately to modified AC11.1 medium with 2 mM BES. Balch tubes were then pressurized with H_2_:CO_2_ (4:1) to 150 kPa and incubated horizontally at 39°C. After 2 days of growth 0.1 ml of each culture was pooled and used as inoculum into 9.9 ml of fresh medium in triplicate and incubated as previously. Headspace gas pressures in the cultures were measured regularly using a pressure gauge (Rydalmere, New South Wales, Australia) with a Luer Lock connecter for needle attachment. Gas was refilled as necessary and cultures were grown in this manner for a maximum of five weeks to enrich for hydrogen utilisers. The ratio of gases in the headspace and concentration of SCFA and sulphide ion in the culture supernatant were determined at harvesting.

### Chemical analyses

Analysis of headspace gas for hydrogen, carbon dioxide and methane was done by gas chromatography on a Shimadzu GC-2014 (Shimadzu, Kyoto, Japan) fitted with a packed Hayesep Q column (1.8 m × 2.00 mm ID, Valco Instruments Co. Inc., Houston, TX). Nitrogen was the carrier gas at 25 ml min^−1^ and separation of gases was achieved over a 3 minute period. The temperature of the column oven and the thermal conductivity detector were 38°C and 100°C respectively.

Concentrations of the SCFAs - acetic, propionic, n-butyric, *iso*butyric, *iso*valeric and n-valeric acid were determined by flame ionization detection on a Shimadzu GC-2014 with a Zebron™ ZB-FFAP column (30 m x 0.53 mm ID, Phenomenex, Torrance, CA), after acidification with ortho-phosphoric acid and with 4 methyl valeric acid at a final concentration of 1 mM as an internal standard. A 0.5 μl aliquot of each sample was injected; the carrier gas was hydrogen at 5 ml min^−1^ and separation of the acids was achieved over 13 minutes. The injector and detector temperature were 200°C and 230°C respectively; the column temperature was initially 100°C for 2 minutes followed by a gradient 15°C min ^−1^ to 230°C with a 2 minute hold. Peak detection and chromatogram integration were performed using GCsolution v 3.30.00 (Shimadzu).

Dissolved sulphide was detected by ion chromatography using the method of Keller-Lehman et al. [29].

An UltiMate® 3000 HPLC system (Dionex, Sunnyvale, CA) with a dedicated Photodiode Array Detector and an Autosampler was used to determine the presence of formic, lactic, acetic, citric, succinic and fumaric acids in culture supernatant. A 2.5 μl aliquot of each sample was injected. Separation and identification was achieved using an Acclaim^TM^ Organic Acid (OA) Analytical Column (5 μm, 4.0 x 150 mm, Dionex) at 30°C using a mobile phase of 50 mM NaH_2_PO_4_ (pH 2.7) at 0.6 ml min^−1^ for 7 minutes. Peak analysis was performed using the Chromeleon software (Dionex).

Glycerol and its derivative 1,3-propanediol were analysed by HPLC as described by Keller-Lehman (pers. comm.). Briefly, 20 μl of each sample was injected on a Phenomenex Rezex ROA-Organic acid H+ column (7.8 x 300 mm) using 0.008 N H_2_SO_4_ as mobile phase at 0.6 ml min^−1^ and 35°C column temperature. A Shimadzu 10A HPLC system with an autoinjector and a degasser was used; samples were detected with a refractive index detector (RID-10A) and analysed using CLASS VP software (Shimadzu).

Potential alcohols produced from glycerol fermentation (ethanol, propanol and n-butanol) were analysed on an Agilent 7890A GC (Agilent Technologies) using a capillary column, DB-FFAP (Agilent, 15 m length x 0.53 mm ID x 1.0 μm film) and an FID detector; high-purity helium with an initial flow of 12.5 ml min^−1^ was used as carrier gas. A 0.5 μl aliquot of each sample was injected at 220°C injection port temperature; the column temperature was 60°C with a hold for 2 minutes, followed by a gradient of 20°C min^−1^ to 240°C (2 minute hold) with a run time of 13 minutes. The FID detector temperature was at 250°C and analysis was done on Agilent GC ChemStation software.

### DNA extraction and molecular analyses of *rrs, acsB and fhs*

DNA was extracted from enrichment cultures using the cetyltrimethylammonium bromide (CTAB) method of Brookman et al. [30] with minor modifications as described previously [8]. Partial *rrs* sequences were amplified using primers 27f and 1492r [31] and PCR, cloning and sequencing was performed as outlined previously [32]. Potentially chimeric sequences were determined using the chimera detection program at the Ribosomal Database Project II [33] and sequences shorter than 500-bp were removed from further analysis. Partial *rrs* sequences were aligned using the NAST aligner and classified using the Hugenholtz taxonomy at the Greengenes database [34]. Sequences were grouped into Operational Taxonomic Units (OTUs) using MOTHUR [35] and a distance ≤ 0.025 to group sequences at an approximate species level [36]. Partial *rrs* sequences (n = 96) from tammar wallaby forestomach enrichment cultures have been submitted to the GenBank [KF264051 to KF264147].

Functional gene (formyltetrahydrofolate synthetase, *fhs* and acetyl-CoA synthase, *acsB*) based analyses of acetogens were performed using partial *acsB* amplified from enrichment culture DNA using the primers ACS_f and ACS_r and protocol of Gagen et al. [8]. Amplicons were cloned as above and sequenced with vector primers T7 and SP6 (Promega). Partial *fhs* were amplified using the primers and protocol of Leaphart & Lovell [37] and clone libraries were constructed as for *acsB* sequences. Deduced amino acids of *acsB* (ACS) and of the formyl-tetrahydrofolate synthetase (*fhs*) (FTHFS) were aligned in ARB [38] with publicly available sequences and grouped into Operational Taxonomic Units (OTUs) at a distance of ≤ 0.035 for ACS and ≤ 0.025 for FTHFS, to cluster similar sequences while separating those from distinct species [8]. Maximum likelihood trees of deduced ACS or FTHFS amino acid sequences were constructed as outlined previously [8] using RaxML [39] at the CIPRES portal [40] and the Jones Taylor Thornton [41] model of amino acid substitutions with a gamma rate of substitution and 25 discrete rate categories. Bootstrap analysis was performed for the best-scoring tree topology with 100 resamplings, using RaxML [39]. FTHFS sequences were assessed for similarity to FTHFSs from authentic acetogens using the HS score of Henderson et al. [42]. Putative *fhs* sequences recovered in this study (n = 64) have been submitted to GenBank [JN1972107 to JN197271]. Putative *acsB* sequences recovered in this study (n = 41) have been submitted to GenBank [JN197042 to JN197082].

### Isolation of acetogens from enrichment cultures

Isolation of potential acetogens (heterotrophic/autotrophic) was carried out in an anaerobic chamber of atmosphere CO_2_:H_2_ (95:5) by a 10-fold serial dilution of actively growing enrichment cultures to 10^−8^. To a 2 ml aliquot of each of these dilutions an equal volume of 2% low melting point agarose in modified AC11.1 containing 2.5 g of glucose, 2.5 g of cellobiose was added at approximately 40°C. This agarose/bacterial mix was poured as an overlay onto modified AC11.1 agar plates and allowed to solidify before being incubated at 39°C in a 3.4 L anaerobic jar (Oxoid Ltd, Cambridge, UK) with the atmosphere of the anaerobic chamber before addition of H_2_ to 30 kPa overpressure. Glucose and cellobiose were included in the agar overlay to stimulate growth and aid colony formation of acetogens that had been enriched on the previous, limiting medium; few colonies formed on solid medium without added organics.

Single colonies that appeared on or in the agar after one week were picked in an anaerobic hood (atmosphere as above) into 1 ml of liquid modified AC11.1 medium in 1.6 ml round 96-well deep well plates (Axygen, Union City, CA). The 96-well deep well plates were sealed with Airpore™ (Qiagen, Hilden, Germany) plate sealer and incubated at 39°C inside the anaerobic chamber. After three days, cultures from this original plate were inoculated (1:50) into fresh medium in 96-well deep well plates as replicates and grown as before. Crude DNA was extracted from the original plate by the following method: the 96-well deep well plates were centrifuged at 6000 *g* for 20 minutes at 4°C and the supernatant discarded; approximately 200 mg of a 1:1 mixture of 0.1 mm and 1.0 mm silica-zirconium beads (Biospec, Bartlesville, OK) and 1 ml of TE buffer (10 mM Tris, 1 mM EDTA) were added to each well. Samples were homogenized in a Retsch® Mixer Mill MM 300 (Retsch, Haan, Germany) fitted with an adapter for 96-well plates (Qiagen) at maximum speed for 2 minutes. After centrifugation, 100 μl of the supernatant containing DNA was transferred to a new 96-well PCR plate, heated at 80°C for 20 minutes and 1 μl of this lysate used in a PCR to screen for the presence of the *fhs* gene using the primers and protocol of Leaphart & Lovell [37] at a modified annealing temperature of 52°C. Cultures from the replicate 96-well deep wells corresponding to *fhs* positive PCRs were inoculated into modified AC11.1 medium in Balch tubes, pressurised with 150 kPa 4:1 H_2_:CO_2_ and monitored for hydrogen consumption.

### Preliminary investigation of tammar wallaby forestomach acetogen

Potential acetogens from the tammar wallaby forestomach enrichment cultures were further purified by streaking on agar plates and picking single colonies. Three potential acetogen isolates obtained were almost identical at the *rrs* level, therefore detailed analyses were only performed on one isolate, arbitrarily named isolate TWA4. Isolate TWA4 was grown horizontally in triplicate at 39°C in modified AC11.1 medium with vitamins (vitamin solution from the DSMZ Medium 141 http://www.dsmz.de/microorganisms/medium/pdf/DSMZ_Medium141.pdf) under 150 kPa 4:1 H_2_:CO_2_ and tubes laid horizontally with gentle shaking at 40 rpm on a gyratory shaker. The purity of isolate TWA4 was checked by microscopy and confirmed by the absence of any mixed chromatograms peaks when the 27f/1492r 16S rDNA PCR product was directly sequenced with each of the primers 27f, 519r, 530f, 907r and 1492r [31]. Hydrogen utilisation, SCFA and organic acid production were determined by GC and HPLC as outlined above. DNA from isolate TWA4 cultures was extracted as outlined above and *rrs, fhs* and *acsB* were amplified and analysed as for enrichment cultures. Additionally, longer *acsB* fragments from isolate TWA4 were obtained by PCR with primer combinations ACSF1 with ACSR1 [8] and ACS851F (5′-GGMTTCCCDGYHRTNACHRA-3′) with ACS_r; the latter primer pair was used with a final concentration of 0.4 mg ml^−1^ bovine serum albumin (New England Biolabs, Ipswich, MA) in PCR as follows: initial denaturation at 95°C for 5 min; 35 cycles of denaturation at 95°C for 30 s; annealing at 48°C for 30 s; and extension at 72°C for 1 min and 30 s; final extension at 72°C for 7 min. the *fhs*, *acsB* and *rrs* sequences from isolate TWA4 have been deposited at GenBank [JN197085-JN197086, JN197083-JN197084 and JN196964 respectively].

### Microscopic analyses

Isolate TWA4 cell morphology was investigated by phase-contrast and electron microscopy. The Gram reaction of isolate TWA4 was determined by conventional Gram staining [43] and the KOH test [44]. The structure of the cell wall of isolate TWA4 was determined by transmission electron microscopy (TEM) after high pressure freezing and freeze substitution in osmium tetroxide-uranyl acetate in acetone as outlined by McDonald and Webb [45]. Thin sections were stained with uranyl acetate and lead citrate [46] and visualised using a JEOL 1010 transmission electron microscope (Jeol Ltd., Tokyo, Japan).

### Investigation of mixotrophic abilities of isolate TWA4

In preliminary investigations a range of carbon sources and electron acceptors were tested as additives to modified AC11.1 medium with H_2_:CO_2_ to determine whether isolate TWA4 was capable of mixotrophic growth. Early indications were that glycerol appeared to stimulate acetate production and cell densities of isolate TWA4 while hydrogen consumption continued, therefore this substance was investigated in more detail. Isolate TWA4 was grown in triplicate as 100 ml cultures in 185 ml bottles on modified AC11.1 medium with (i) H_2_:CO_2_ (4:1, 145 kPa) (ii) glycerol (10 mM) and H_2_:CO_2_ (4:1, 145 kPa) or (iii) glycerol (10 mM) alone (with the headspace atmosphere as remaining after dispensing in an anaerobic chamber). Headspace gas pressures were monitored by pressure sensors and hydrogen and SCFA concentrations were determined at time zero and every six hours for up to 54 hours.

### Co-culture of isolate TWA4 with a methanogen

In order to investigate the hydrogen threshold and hydrogenotrophic ability of isolate TWA4 in comparison with a methanogen, co-culture experiments were performed with isolate TWA4 and *M. smithii* PS (ATCC 35061). *M. smithii* was chosen from a panel of methanogens for its ability to grow at the same temperature, a similar growth rate and in the same medium as isolate TWA4. With the addition of a further 1.5 g L^−1^ yeast extract to modified AC11.1 medium both isolate TWA4 and *M. smithii* grew robustly; this modified medium used in co-culture experiments is referred to as modified AC11.1Y medium. Isolate TWA4 and *M. smithii* were grown separately on modified AC11.1Y medium to an OD_600_ of 0.2, or diluted to this OD (*M. smithii* only). An equal volume (1:100 each) of these cultures was inoculated, in an anaerobic hood (atmosphere as previously) for co-culture studies. Cultures were performed in triplicate in 10 ml media in 27 ml Balch tubes under autotrophic conditions (i.e. modified AC11.1Y with H_2_:CO_2_ (4:1) at 131.5 kPa) or heterotrophic conditions (modified AC11.1Y medium with glycerol at a final concentration of 10 mM and headspace as per the anaerobic chamber atmopshere) and included (i) isolate TWA4 alone (ii) *M. smithii* alone and (iii) a co-culture of isolate TWA4 and *M. smithii* inoculated together. Triplicates of uninoculated medium served as controls.

### Statistical analyses

A student’s *t*-test was used to compare differences of means. Differences were considered significant at p < 0.05.

### Genome sequencing

Isolate TWA4 was grown in 500 ml of modified AC11.1Y medium with H_2_:CO_2_ (4:1) provided at 150 kPa, in a 1 L anaerobic bottle (Bellco). Cells were harvested (6000 *g*, 15 min, 4°C) after approximately 72 h growth (culture optical density at 600 nm ~0.20) and DNA was extracted by enzymatic lysis as outlined by Pope et al. [47], except also including 60 s bead-beating in a FastPrep®-24 bead-beater (MP Biomedicals, Solon, OH) and incubation at 70°C for 10 minutes prior to chloroform/isoamyl alcohol extraction. The DNA was checked for quality by agarose gel electrophoresis, quantified by Quant-iT^TM^ dsDNA assay kit (Invitrogen, Carlsbad, CA) and sequenced on a Roche/454 FLX sequencer using a shotgun/ 10 kb paired-end sequencing method. Sequence reads were assembled into scaffolds using Newbler version 2.6 and uploaded to the Rapid Annotation using Subsystem Technology (RAST) server for gene calling and annotation [48]. This Whole Genome Shotgun project has been deposited at DDBJ/EMBL/Genbank under the accession JPZU00000000. The version described in this paper is version JPZU01000000.

## Additional files

Additional file 1:
**Net change in gases and short chain fatty acids in tammar wallaby acetogen enrichment cultures.**
Additional file 2:
**Rarefaction analysis of** ***rrs*** **library from tammar wallaby forestomach enrichment cultures.**
Additional file 3:
**Maximum likelihood tree of** ***rrs*** **sequences from isolate TWA4, nearest named isolates and other acetogens.** GenBank accession numbers of reference sequences are shown after species names. Branch nodes with ≥ 75% bootstrap support are marked with closed circles. The scale bar represents 10% sequence divergence. Additional file 4:
**a - Maximum likelihood tree of FTHFS from tammar wallaby forestomach enrichment cultures (TWE) and isolate TWA4.** Tree is of deduced FTHFS amino acid sequences. GenBank accession numbers of reference sequences are shown after species names. Branch nodes with ≥ 75% bootstrap support (100 replicates) are marked with closed circles. The scale bar represents 10% sequence divergence. The number of sequences in OTUs is indicted in brackets. FTHFS HS scores are included as percentages in brackets for OTUs recovered in this study. b - Rarefaction analysis of FTHFS library from tammar wallaby forestomach enrichment cultures. Additional file 5:
**a - Maximum likelihood tree of ACS from tammar wallaby forestomach enrichment cultures (TWE) and isolate TWA4 Tree is of deduced ACS amino acid sequences.** GenBank accession numbers of reference sequences are shown after species names. Branch nodes with ≥ 75% bootstrap support (100 replicates) are marked with closed circles. The scale bar represents 10% sequence divergence. The number of sequences in OTUs or closed groups is indicted in brackets.b - Rarefaction analysis of ACS library from tammar wallaby forestomach enrichment cultures.
