# Supplementary material for: Investigation of a new acetogen isolated from an enrichment of the tammar wallaby forestomach
Source: BMC Microbiol. 2014 Dec 11;14:314. doi: 10.1186/s12866-014-0314-3 (PMC4275979; doi:10.1186/s12866-014-0314-3)
Supplement: Additional file 1: — Net change in gases and short chain fatty acids in tammar wallaby acetogen enrichment cultures. [file 12866_2014_314_MOESM1_ESM.docx]

**Additional file 1 - Net change in gases, short chain fatty acids and sulphide in tammar wallaby acetogen enrichment cultures.**

| **Compound** | **Net change (μmoles) over four weeks** |
| --- | --- |
| H_2_ | -2383.2 ± 268.3 |
| CO_2_ | -259.1 ± 84.1 |
| CH_4_ | not detected |
| acetic acid | 367.7 ± 54.9 |
| propionic acid | -0.8 ± 1.1 |
| iso-butyric acid | 0.6 ± 0.3 |
| n-butyric acid | 11.5 ± 3.3 |
| iso-valeric acid | 0.8 ± 0.5 |
| n-valeric acid | -0.8 ± 0.2 |
| sulphide ion | 2.8 ± 2.0 |
