# Supplementary figures and images for: Investigation of a new acetogen isolated from an enrichment of the tammar wallaby forestomach
Source: BMC Microbiol. 2014 Dec 11;14:314. doi: 10.1186/s12866-014-0314-3 (PMC4275979; doi:10.1186/s12866-014-0314-3)

## Slide 1
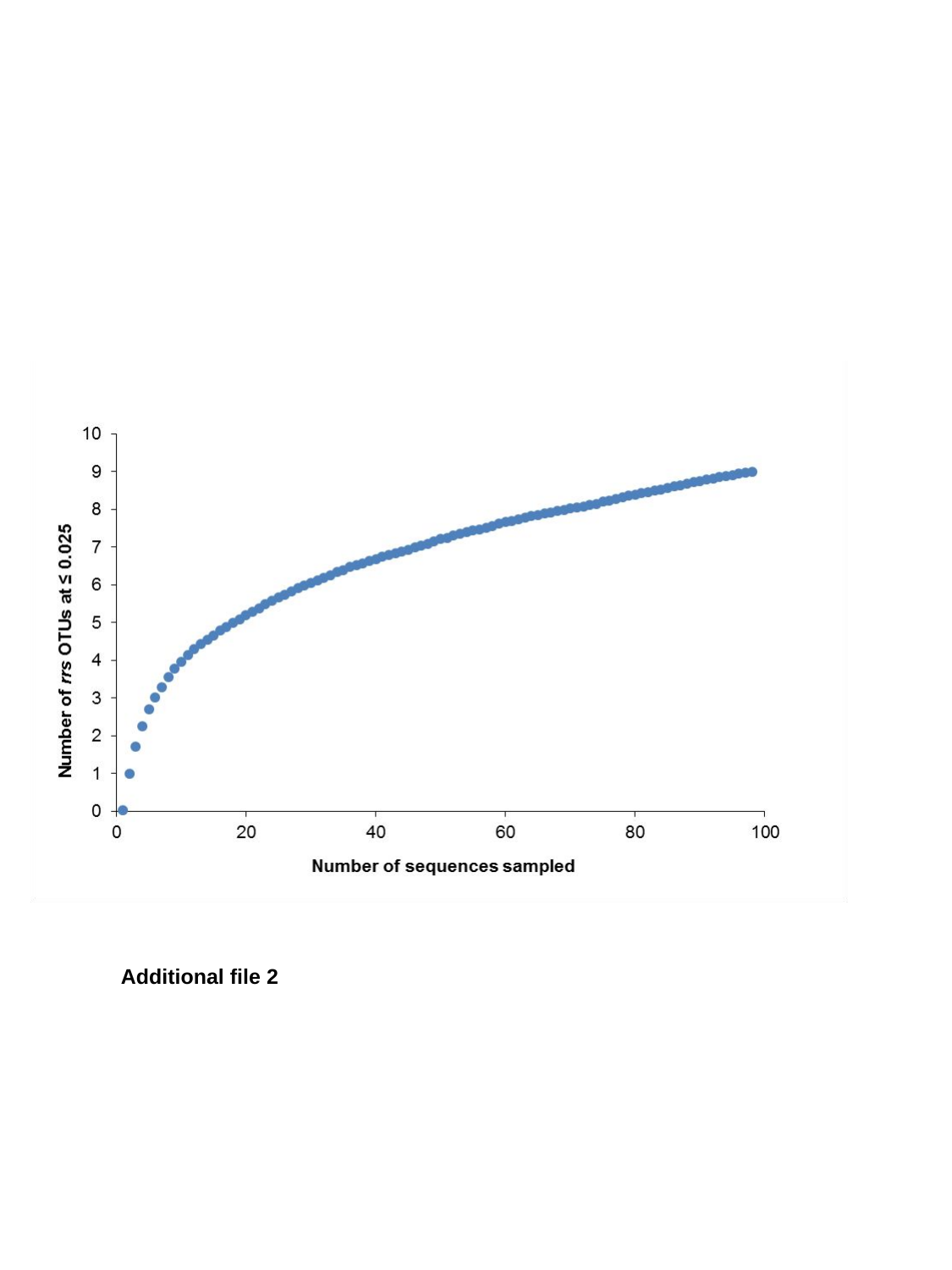

Additional file 2

Supplement: Additional file 2: — Rarefaction analysis of rrs library from tammar wallaby forestomach enrichment cultures. [file 12866_2014_314_MOESM2_ESM.pptx]
