# Supplementary material for: Investigation of a new acetogen isolated from an enrichment of the tammar wallaby forestomach
Source: BMC Microbiol. 2014 Dec 11;14:314. doi: 10.1186/s12866-014-0314-3 (PMC4275979; doi:10.1186/s12866-014-0314-3)
Supplement: Additional file 3: — Maximum likelihood tree of rrs sequences from isolate TWA4, nearest named isolates and other acetogens. GenBank accession numbers of reference sequences are shown after species names. Branch nodes with ≥ 75% bootstrap support are marked with closed circles. The scale bar represents 10% sequence divergence. [file 12866_2014_314_MOESM3_ESM.pptx]

## Slide 1
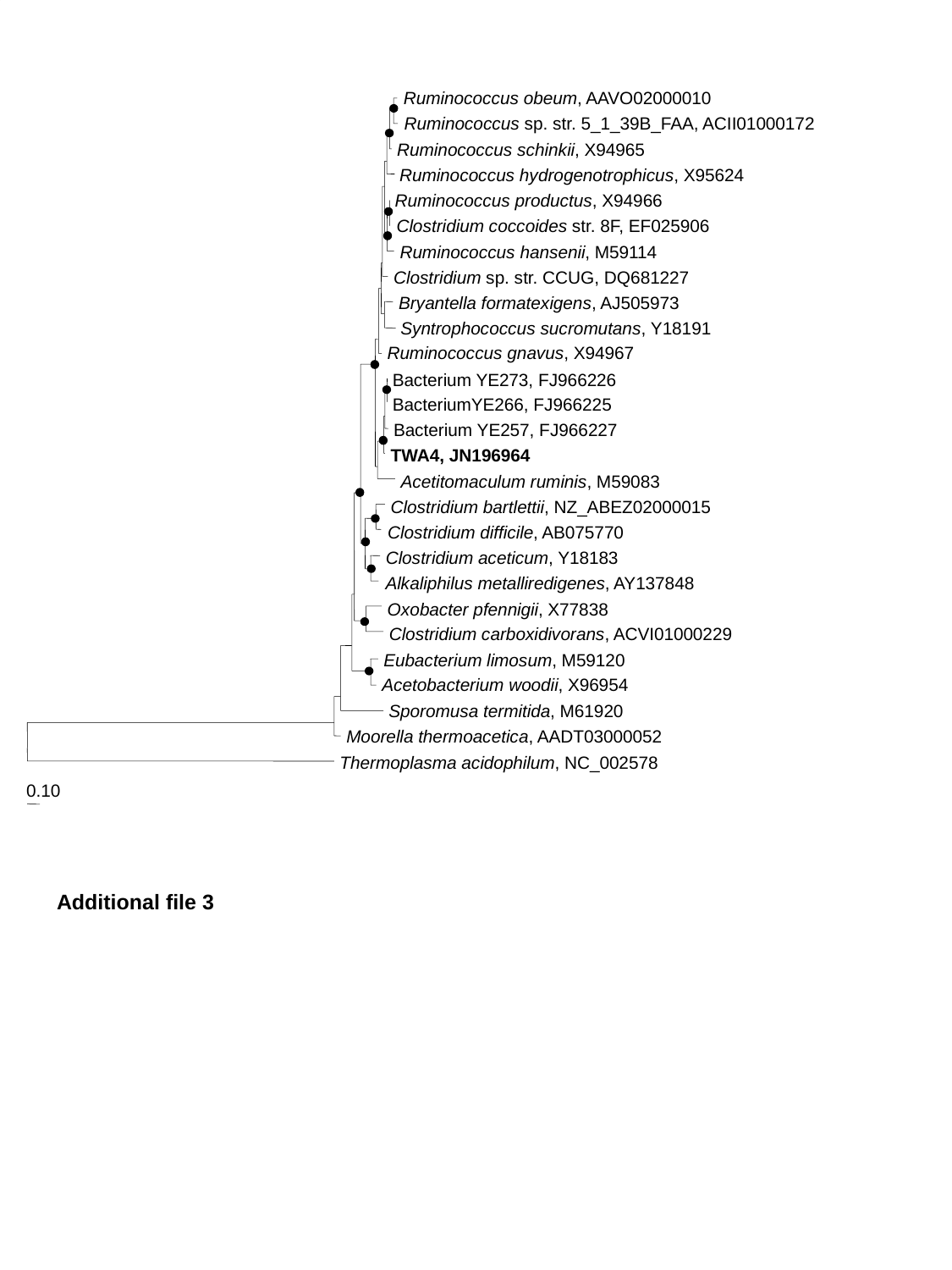

Ruminococcus obeum, AAVO02000010
Ruminococcus sp. str. 5_1_39B_FAA, ACII01000172
Ruminococcus schinkii, X94965
Ruminococcus hydrogenotrophicus, X95624
Ruminococcus productus, X94966
Clostridium coccoides str. 8F, EF025906
Ruminococcus hansenii, M59114
Clostridium sp. str. CCUG, DQ681227
Bryantella formatexigens, AJ505973
Syntrophococcus sucromutans, Y18191
Ruminococcus gnavus, X94967
Bacterium YE273, FJ966226
BacteriumYE266, FJ966225
Bacterium YE257, FJ966227
TWA4, JN196964
Acetitomaculum ruminis, M59083
Clostridium bartlettii, NZ_ABEZ02000015
Clostridium difficile, AB075770
Clostridium aceticum, Y18183
Alkaliphilus metalliredigenes, AY137848
Oxobacter pfennigii, X77838
Clostridium carboxidivorans, ACVI01000229
Eubacterium limosum, M59120
Acetobacterium woodii, X96954
Sporomusa termitida, M61920
Moorella thermoacetica, AADT03000052
Thermoplasma acidophilum, NC_002578
0.10
Additional file 3
