# Supplementary material for: Investigation of a new acetogen isolated from an enrichment of the tammar wallaby forestomach
Source: BMC Microbiol. 2014 Dec 11;14:314. doi: 10.1186/s12866-014-0314-3 (PMC4275979; doi:10.1186/s12866-014-0314-3)
Supplement: Additional file 4: — a - Maximum likelihood tree of FTHFS from tammar wallaby forestomach enrichment cultures (TWE) and isolate TWA4. Tree is of deduced FTHFS amino acid sequences. GenBank accession numbers of reference sequences are shown after species names. Branch nodes with ≥ 75% bootstrap support (100 replicates) are marked with closed circles. The scale bar represents 10% sequence divergence. The number of sequences in OTUs is indicted in brackets. FTHFS HS scores are included as percentages in brackets for OTUs recovered in this study. b - Rarefaction analysis of FTHFS library from tammar wallaby forestomach enrichment cultures. [file 12866_2014_314_MOESM4_ESM.pptx]

## Slide 1
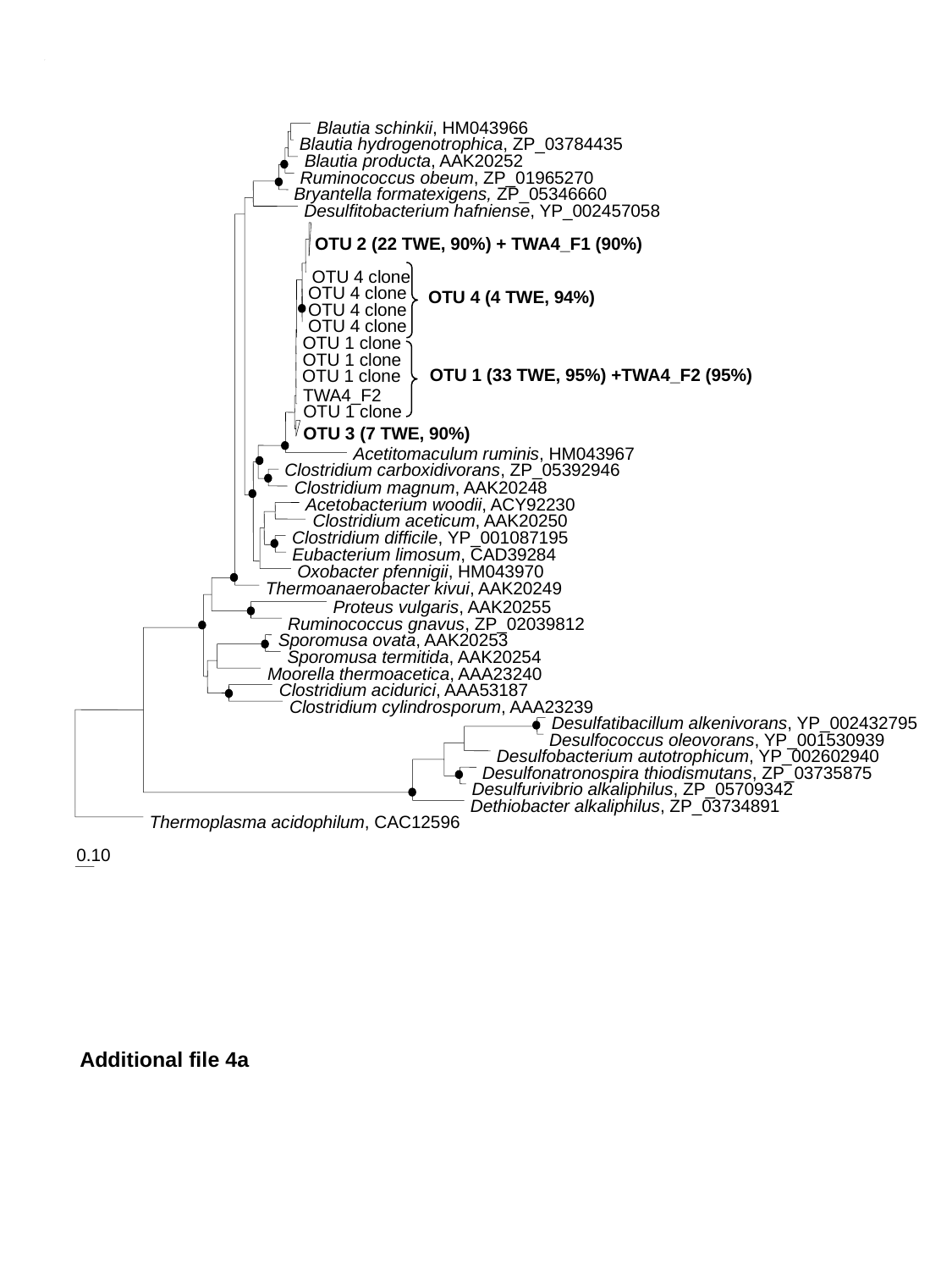

Blautia schinkii, HM043966
Blautia hydrogenotrophica, ZP_03784435
Blautia producta, AAK20252
Ruminococcus obeum, ZP_01965270
Bryantella formatexigens, ZP_05346660
Desulfitobacterium hafniense, YP_002457058
OTU 2 (22 TWE, 90%) + TWA4_F1 (90%)
OTU 4 clone
OTU 4 clone
OTU 4 (4 TWE, 94%)
OTU 4 clone
OTU 4 clone
OTU 1 clone
OTU 1 clone
OTU 1 (33 TWE, 95%) +TWA4_F2 (95%)
OTU 1 clone
TWA4_F2
OTU 1 clone
OTU 3 (7 TWE, 90%)
Acetitomaculum ruminis, HM043967
Clostridium carboxidivorans, ZP_05392946
Clostridium magnum, AAK20248
Acetobacterium woodii, ACY92230
Clostridium aceticum, AAK20250
Clostridium difficile, YP_001087195
Eubacterium limosum, CAD39284
Oxobacter pfennigii, HM043970
Thermoanaerobacter kivui, AAK20249
Proteus vulgaris, AAK20255
Ruminococcus gnavus, ZP_02039812
Sporomusa ovata, AAK20253
Sporomusa termitida, AAK20254
Moorella thermoacetica, AAA23240
Clostridium acidurici, AAA53187
Clostridium cylindrosporum, AAA23239
Desulfatibacillum alkenivorans, YP_002432795
Desulfococcus oleovorans, YP_001530939
Desulfobacterium autotrophicum, YP_002602940
Desulfonatronospira thiodismutans, ZP_03735875
Desulfurivibrio alkaliphilus, ZP_05709342
Dethiobacter alkaliphilus, ZP_03734891
Thermoplasma acidophilum, CAC12596
0.10
Additional file 4a

## Slide 2
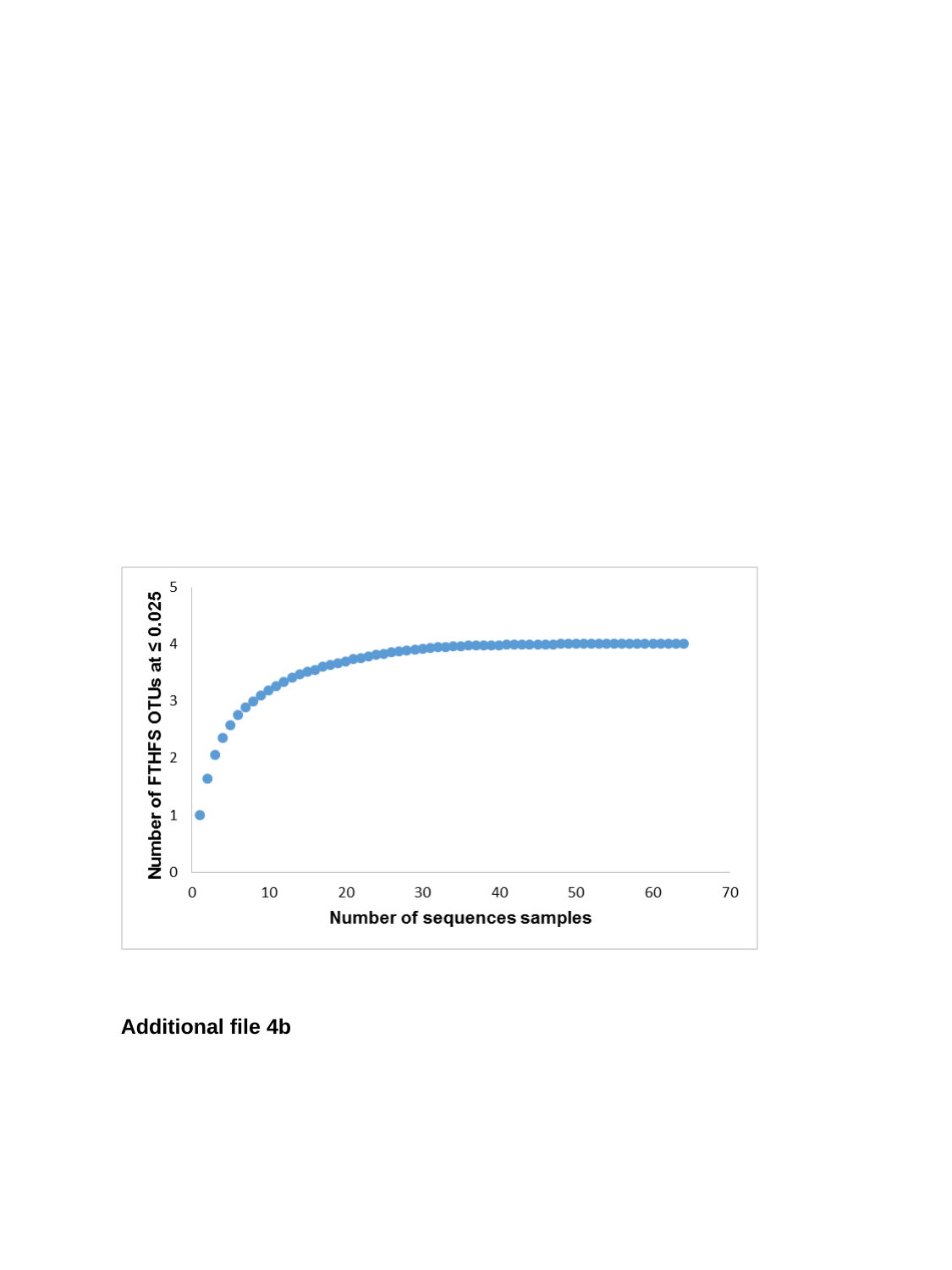

Additional file 4b
