# Supplementary material for: Investigation of a new acetogen isolated from an enrichment of the tammar wallaby forestomach
Source: BMC Microbiol. 2014 Dec 11;14:314. doi: 10.1186/s12866-014-0314-3 (PMC4275979; doi:10.1186/s12866-014-0314-3)
Supplement: Additional file 5: — a - Maximum likelihood tree of ACS from tammar wallaby forestomach enrichment cultures (TWE) and isolate TWA4 Tree is of deduced ACS amino acid sequences. GenBank accession numbers of reference sequences are shown after species names. Branch nodes with ≥ 75% bootstrap support (100 replicates) are marked with closed circles. The scale bar represents 10% sequence divergence. The number of sequences in OTUs or closed groups is indicted in brackets.b - Rarefaction analysis of ACS library from tammar wallaby forestomach enrichment cultures. [file 12866_2014_314_MOESM5_ESM.pptx]

## Slide 1
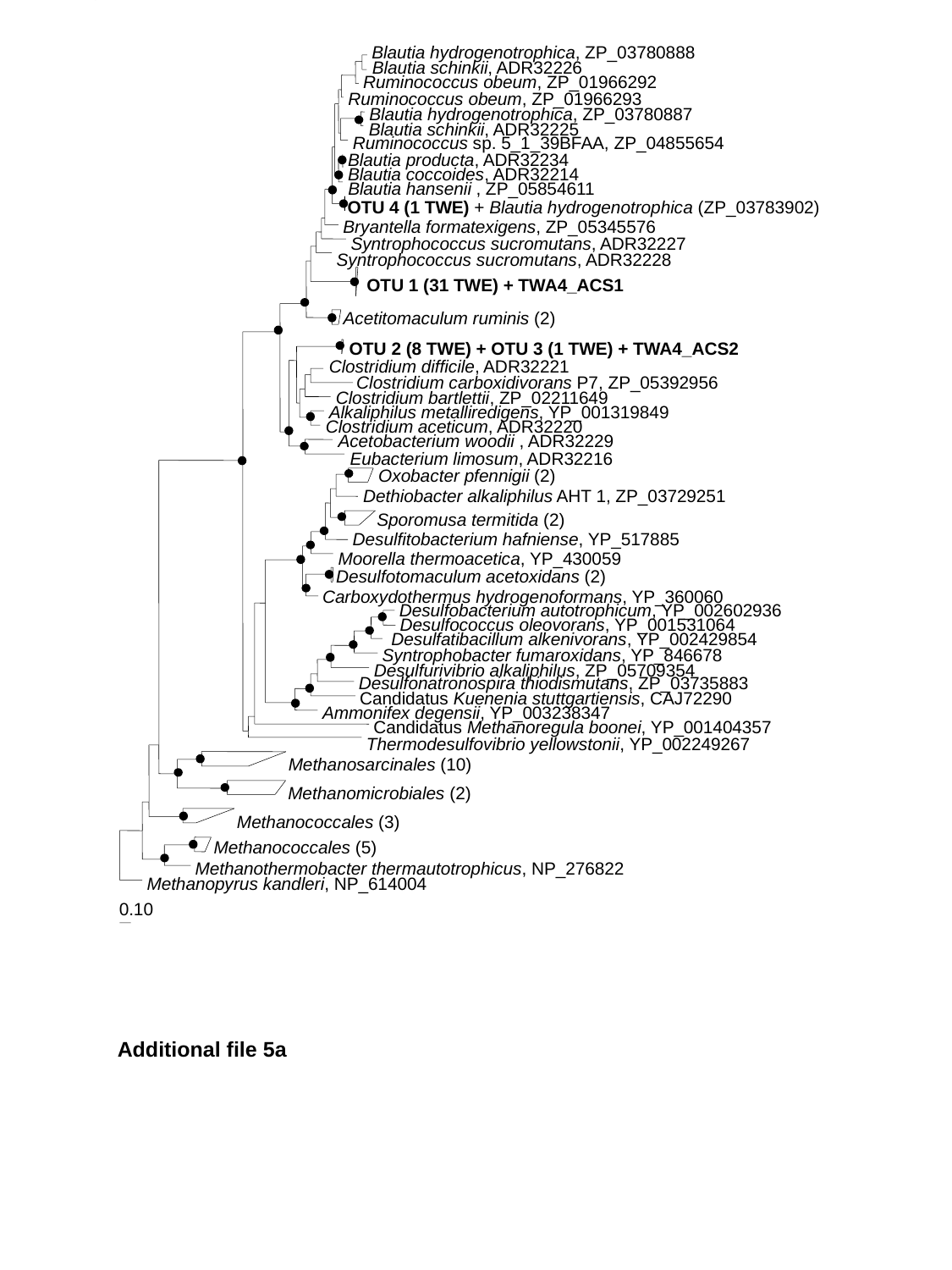

Blautia hydrogenotrophica, ZP_03780888
Blautia schinkii, ADR32226
Ruminococcus obeum, ZP_01966292
Ruminococcus obeum, ZP_01966293
Blautia hydrogenotrophica, ZP_03780887
Blautia schinkii, ADR32225
Ruminococcus sp. 5_1_39BFAA, ZP_04855654
Blautia producta, ADR32234
Blautia coccoides, ADR32214
Blautia hansenii , ZP_05854611
OTU 4 (1 TWE) + Blautia hydrogenotrophica (ZP_03783902)
Bryantella formatexigens, ZP_05345576
Syntrophococcus sucromutans, ADR32227
Syntrophococcus sucromutans, ADR32228
OTU 1 (31 TWE) + TWA4_ACS1
Acetitomaculum ruminis (2)
OTU 2 (8 TWE) + OTU 3 (1 TWE) + TWA4_ACS2
Clostridium difficile, ADR32221
Clostridium carboxidivorans P7, ZP_05392956
Clostridium bartlettii, ZP_02211649
Alkaliphilus metalliredigens, YP_001319849
Clostridium aceticum, ADR32220
Acetobacterium woodii , ADR32229
Eubacterium limosum, ADR32216
Oxobacter pfennigii (2)
Dethiobacter alkaliphilus AHT 1, ZP_03729251
Sporomusa termitida (2)
Desulfitobacterium hafniense, YP_517885
Moorella thermoacetica, YP_430059
Desulfotomaculum acetoxidans (2)
Carboxydothermus hydrogenoformans, YP_360060
Desulfobacterium autotrophicum, YP_002602936
Desulfococcus oleovorans, YP_001531064
Desulfatibacillum alkenivorans, YP_002429854
Syntrophobacter fumaroxidans, YP_846678
Desulfurivibrio alkaliphilus, ZP_05709354
Desulfonatronospira thiodismutans, ZP_03735883
Candidatus Kuenenia stuttgartiensis, CAJ72290
Ammonifex degensii, YP_003238347
Candidatus Methanoregula boonei, YP_001404357
Thermodesulfovibrio yellowstonii, YP_002249267
Methanosarcinales (10)
Methanomicrobiales (2)
Methanococcales (3)
Methanococcales (5)
Methanothermobacter thermautotrophicus, NP_276822
Methanopyrus kandleri, NP_614004
0.10
Additional file 5a

## Slide 2
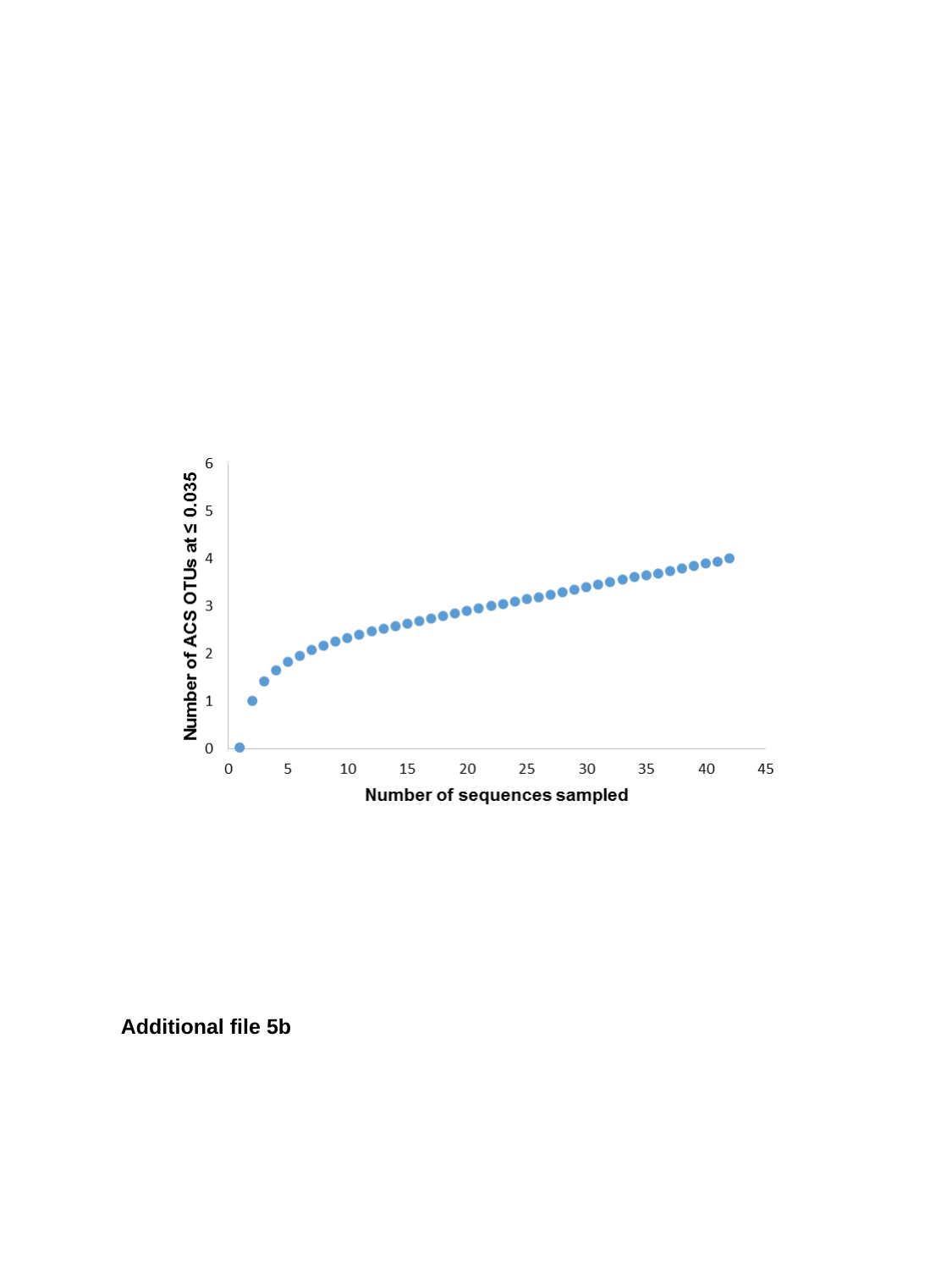

Additional file 5b
